# Analysis of transient otoacoustic emissions and brainstem evoked auditory potentials in neonates with hyperbilirubinemia

**DOI:** 10.1016/S1808-8694(15)30656-X

**Published:** 2015-10-19

**Authors:** Daniela Polo Camargo da Silva, Regina Helena Garcia Martins

**Affiliations:** 1 Speech therapist of the ENT Discipline, Faculdade de Medicina de Botucatu; graduate student in the Bases Gerais da Cirurgia program; 2 Assistant professor, doctor in surgery, Faculdade de Medicina de Botucatu (Unesp). In charge of the Phoniatrics and Voice Clinic. Faculty member of the Otorhinolaryngology Discipline, Universidade Estadual Paulista (Unesp), Botucatu campus. Universidade Estadual Paulista (Unesp), Faculdade de Medicina, Campos de Botucatu

**Keywords:** hyperbilirubinemia, neonatal, hearing loss

## Abstract

Hyperbilirubinemia is toxic to the auditory pathways and to the central nervous system, leaving sequelae such as hearing loss and encephalopathy.

**Aim:**

to assess the hearing of neonates with hyperbilirubinemia, using transient evoked otoacoustic emissions (TOAEs) and brainstem evoked auditory potentials (BEAP). Prospective study.

**Materials and Methods:**

we had two groups: GI (n-25), neonates with hyperbilirubinemia; GII (n-22), neonates without hyperbilirubinemia and without risk factors for hearing loss. All the neonates had up to 60 days of life and were submitted to TOAE and BEAP.

**Results:**

12 neonates from GI and 10 from GII were girls and 13 from GI and 12 from GII were boys. TOAEs were present in all the children, however with lower amplitudes in GI, especially in the frequencies of 2 and 3KHz (p < 0.05). Regarding the BEAP, we observed a mild PV and LI-V increase in BI. The alterations observed in these tests do not correlate to the serum levels of bilirubin.

**Conclusions:**

in neonates with hyperbilirubinemia, we noticed lower TOAE amplitudes and mild PV and LI-V increase, indicating cochlear and retrocochlear disorders, stressing the importance of using both tests and carefully reading them in these evaluations.

## INTRODUCTION

Hearing is responsible for the development of spoken language, which requires an intact peripheral and central auditory system. Many factors may affect hearing during the neonatal period, including hyperbilirubinemia, which manifests clinically as jaundice.

Jaundice is defined as a yellowish skin color due to increased bilirubin levels in extracellular fluids. It is considered physiological when increased non-conjugated bilirubin levels occur within the first two weeks of life. Pathological cases that cause concern present elevated levels of this pigment within the first 24 hours of life; early treatment is required, whether phototherapy or exchange transfusion.^1^,^2^

Many authors have highlighted the harmful effects of biliary pigment impregnation of central and peripheral auditory pathways.^3^, ^4^, ^5^ Hyperbilirubinemia is toxic for endocochlear hair cells, basal nuclei and central auditory pathways.^4^ The intensity of hearing loss may vary and it may or may not be reversible. An early diagnosis - within the first months of life - is mandatory. Testing of otoacoustic emissions (OAE) and brainstem auditory evoked potentials (BAEP) stand out among the diagnostic methods due to their objectivity and reliability. OAE assess cochlear function, specifically that of the outer hair cells (preneural pathways), and are used in assessing cochlear diseases, such as exposure to ototoxic substances, viruses, and noise; it is also applied in neonatal auditory screening.^6^, ^7^, ^8^ BAEP record the electrophysiological activity of the peripheral auditory system up to the brainstem, which arises within the first 10-12 ms following sound stimulation; retrocochlear pathways may thus be studied.^9^

In neonates with hyperbilirubinemia, evoked OAE (EOAE) may or may not yield responses; the latter indicates cochlear damage. BAEP may detect an increased electrophysiological threshold, absence of responses, and prolonged wave and interpeak absolute latencies; these changes may or may not be reversible with therapy. According to some authors, therapeutic success is related with serum bilirubin levels.^4^,^10^, ^11^, ^12^ On the other hand, other authors have not demonstrated altered auditory pathways in neonates with hyperbilirubinemia, such as in _gun et al.'s13 study of 30 newborns aged from 24 to 72 months, who were screened with BAEP, transient-evoked OAE (TEOAE) and a speech-language questionnaire. OAE and electrophysiological potentials recorded at 80 dB were present in all children, who also had normal language development. These authors found no correlation among total serum bilirubin levels and altered BAEP thresholds and latencies.

These contradictory results justify new studies to assess auditory pathway integrity in neonates with hyperbilirubinemia to establish the degree of harm and the sectors affected.

## SERIES AND METHOD

### Series

Two study groups were defined: a study or sample group (GI) consisting of 25 neonates with hyperbilirubinemia, confirmed as serum indirect bilirubin levels above 3 mg/dL, and undergoing phototherapy and/or exchange transfusion; a control group (GII) composed of 22 neonates with serum indirect bilirubin levels below 3 mg/dL and no risk factors for hearing loss. All control group neonates underwent the same sequence of auditory assessments as the study group.

The caretakers of candidates for the study and control groups signed a free informed consent form. The Research Ethics Committee of the institution in which research was conducted approved the study (protocol 271/2007).

The following exclusion criteria were defined for standardization purposes: a family history of hearing loss (including reports of inherited or genetic deafness); use of ototoxic drugs by mothers during pregnancy or by neonates; presence of cranio-facial malformations; altered otoscopy; neonatal hypoxia (assessed as Apgar values below 4 in the first minute and 6 in the fifth minute); aged above three months.

## METHOD

Initially, parents were interviewed to fill in the study protocol and to gather a clinical history. A review was made of medical files in search of data on: age, sex, intercurrences during pregnancy, birth status, blood type of the mother and of the newborn, serum direct and indirect bilirubin levels; and type of therapy (phototherapy and/or exchange transfusion).

Auditory assessment - prior to an evaluation of hearing, all neonates underwent meatoscopy, which was carried out by an otorhinolaryngologist using a battery-operated Heyne otoscope (Germany).

Audiological studies consisted of TEOAE and BAEP testing. TEOAE were tested with non-linear clicks, in an acoustic booth using a cochlear emissions analyzer (ILO 288, Otodynamic Ltda) coupled to a microcomputer. The Quickscreener software, indicated for auditory screening, was used in the procedure. Responses were recorded at frequency bands of 1.0, 1.5, 2.0, 3.0 and 4.0 Hz within a 12 ms window. The following analytical parameters were used for interpreting the results: probe stability above 70%, stimulus intensity from 79 to 83 dB, signal reproducibility over 70%, and response amplitude equal to or above 6 dBSPL over the noise spectrum in three consecutive frequencies.

BAEP testing was done using an EP-15 device (Eclipse, Interacoustics, Denmark) in a silent room. After skin cleaning with an abrasive substance, positive or active surface electrodes (Neuroline) were placed on the forehead, and negative or reference electrodes were placed on the mastoid area. The ground electrode was placed on the forehead. Monaural stimuli (80 dBSPL rarefied polarity 100 ms filtered clicks from 100 to 3,000 Hz) were presented through insertion earphones (ER 3A). There were 1,024 clicks with a 15 ms analysis time, repeated to confirm wave reproduction. The electrode impedance was always below 5 KOhms. The stimulus frequency was 20.1 clicks per second.

Pearson's correlation coefficient was applied in the statistical analysis for measuring associations among variables;14 the significance level was 5%. Statistically significant results are marked (*) in the Tables.

## RESULTS

**Figure 1:**
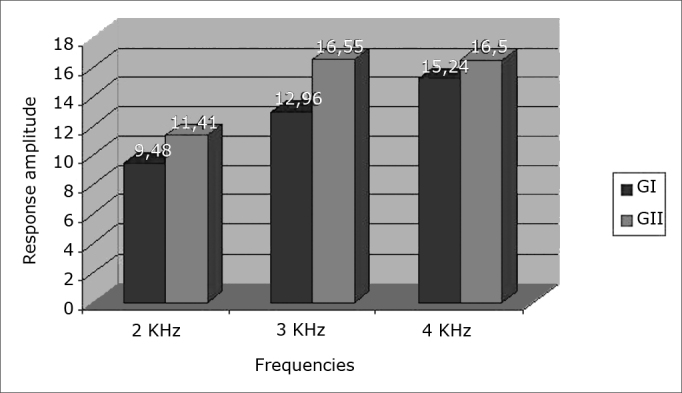
Response amplitude at 2K, 3K and 4KHz frequency ranges in transient otoacoustic emissions testing in the right ear in both study groups.

**Figure 2:**
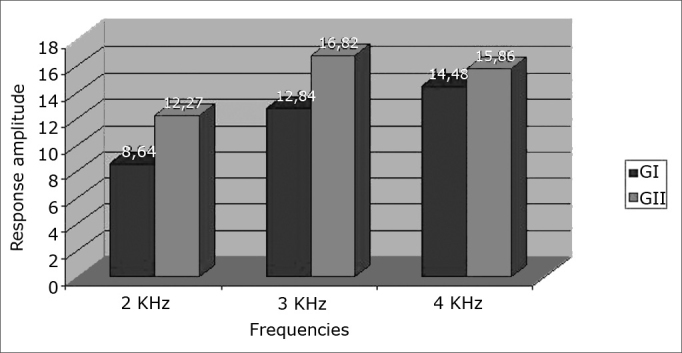
Response amplitude at 2K, 3K and 4KHz frequency ranges in transient otoacoustic emissions testing in the left ear in both study groups.
  Seven neonates in GI had serum unconjugated bilirubin levels over 15mg/dL; four of these neonates had undergone exchange transfusion, and three neonates had undergone repeated phototherapy sessions. OAE amplitude values, especially in these seven children, were not the lowest recorded values, which made it impossible to establish a causal relation among variables.
• BAEP results in ears tested in both groups
  PI and PIII values were similar in both groups. Right ear PV was discretely prolonged in GI (Table 1; p < 0.05) as was LI-V; other interpeaks showed no differences (Table 2; p < 0.05). These recorded BAEP alterations in GI neonates were not matched with the seven children that had the highest serum bilirubin levels.

• Age range
  11 neonates in GI (44%) and 13 neonates in GII (59%) were aged at least one month; 14 neonates in GI (56%) and nine neonates in GII (41%) were aged two months.
• Sex
  There were no sex differences between groups; there were 12 female neonates in GI (48%) and 10 female neonates in GII (45.50%); there were 13 male neonates in GI (52%) and 12 male neonates in GII (54.50%).
• Results of TEOAE responses per frequency band – 2K, 3K and 4KHz - in ears tested in both groups
  Figure 1, Figure 2 show increased response amplitudes as the frequency increases in both groups. Amplitudes in GI were lower than in GII, which was statistically significant at 2 and 3KHz (p < 0.05).

**Table 1:** Mean and standard deviation of absolute latencies of waves PI, PIII and PV in each study group (in ms)

Group	Ear		Wave	
		I	III	V
GI	RE	1,40 ± 0,09	4,13 ± 0,24	6,31 ± 0,26 *
	LE	1,42 ± 0,09	4,14 ± 0,25	6,29 ± 0,26
GII	RE	1,41 ± 0,11	4,02 ± 0,10	6,17 ± 0,16
	LE	1,40 ± 0,10	4,04 ± 0,12	6,22 ± 0,18

**Table 2:** Mean and standard deviation of interpeak latencies LI - III, LIII - V and LI - V in each study group (in ms).

Group	Ear		Interpeaks	
		I - III	III - V	I - V
GI	RE	2,72 ± 0,23	2,18 ± 0,17	4,90 ± 0,26 *
	LE	2,72 ± 0,23	2,14 ± 0,18	4,87 ± 0,26
GII	RE	2,60 ± 0,13	2,15 ± 0,15	4,76 ± 0,20
	LE	2,64 ± 0,16	2,17 ± 0,14	4,81 ± 0,21

## DISCUSSION

Elevated serum levels of unconjugated bilirubin are considered toxic for the auditory pathways and the central nervous system, and are included among the risk factors for neonatal deafness and encephalopathies. A high concentration of biliary pigments is treated with phototherapy and/or exchange transfusion; the latter is reserved for severe cases and those resistant to repeated phototherapy.^1^,^2^

TEOAE have been widely applied in the assessment of hearing in neonates; it is the test of choice for neonatal auditory screening.^3^,^6^,^15^,^16^ In this study, emissions were found in all neonates of both groups; however, control group (GII) neonates had higher recorded amplitude values compared to GI neonates, especially at 2 to 3 KHz. This finding suggests possible endocochlear involvement due to the toxic effect of biliary pigments. Rapid pass-fail type auditory screening methods used at many centers may miss subtle changes in response amplitudes. Some authors have underlined the importance of analyzing TEOAE amplitudes when comparing term and preterm neonate responses. Amplitudes may be decreased in neonates at any risk for hearing loss, such as in premature birth.^17^ This alteration was encountered in this study, especially in GI neonates, even those with mildly elevated serum biliary pigment. A review of the literature found no studies that analyzed OAE amplitudes in children with hyperbilirubinemia.

It is therefore clear that results should not be interpreted based only on present or absent responses in the auditory evaluation of neonates, but rather on features of the recordings. Minor amplitude variations in OAE may be diagnosed, even when using only TEOAE. However, analyses of distortion product OAE increase the reliability of the method, making it possible to track a wider frequency range.

Martinho & Lewis^5^ and Oysu et al.18 found TEOAE with strong amplitudes in severe hyperbilirubinemia cases; altered BAEP recordings, however, suggested auditory neuropathy.

The results of the present study showed that higher serum bilirubin levels did not alter TOAE amplitude values or BAEP electrophysiological potential latencies. Some authors, however, have presented a cause-effect relation between these variables, as shown by Lenhardt et al.^19^ These authors performed BAEP testing in 10 neonates with hyperbilirubinemia soon after birth and repeated the test in five of these children. They compared the results with a control group consisting of 10 neonates with normal serum bilirubin levels, and found that absolute PIII and PV latencies were higher in the study group, compared with the control group; these values relate directly with increased serum bilirubin concentrations.

The reversibility of auditory pathway damage following the treatment of hyperbilirubinemia could not be established in this study due to delays in referring these children. However, auditory pathway alterations were recorded at two months post-therapy, which suggested temporary or permanent auditory sequelae. Thus it is important to follow up these children on an ambulatory basis, monitoring not only language, but also cognitive development. Wemberg et al.^20^ reported a case of a neonate with hyperbilirubinemia due to Rh incompatibility, in whom BAEP testing was done within 27 hours of life; these authors found that when the serum bilirubin concentration was higher than 15 mg/dL, elevated electrophysiological thresholds and prolonged PV were recorded. The authors repeated BAEP testing after exchange transfusion done at 36 hours of life, and found that electrophysiological thresholds improved, but PV latency remained altered. At three months follow-up, electrophysiological thresholds gradually returned to normal values; however, the LI-V interval remained prolonged, which suggested retrocochlear auditory sequelae.

Nwaesei et al.^21^ also highlighted the reversible nature of auditory alterations after exchange transfusion in a study of nine neonates that were transfused; BAEP testing was done one hour before and one hour after exchange transfusion.

Nakamura et al.^22^ carried out BAEP testing in auditory assessments of 56 hyperbilirubinemic neonates and 24 neonates with no jaundice to verify whether bilirubin could cause early ototoxicity. Absolute PI latencies were increased compared with the control group. These authors also found that isolated wave latencies improved after exchange transfusion, where PI recovered before PV, although the interpeak LI-V latency did not improve.

Sharma et al.^23^ carried out BAEP testing for the auditory assessment of 30 jaundiced neonates soon after birth and after 2 to 4 months. The mean wave absolute latencies and their interpeaks were prolonged in jaundiced neonates compared with controls, suggesting early bilirubin-induced encephalopathy. BAEP alterations persisted in 23.3% of these cases in follow up, demonstrating the importance of this test for early detection of hearing loss.

Long-term follow-up results on auditory pathway integrity vary among authors. Some authors raise the possibility of auditory sequelae even with early and appropriate therapy. Sabatino et al.^24^ applied BAEP testing in the third day of life in 48 term neonates with elevated serum total bilirubin, and repeated the evaluation on the fifth or seventh day after therapy. Supplemental retest recordings were made at three, six and 12 months. The first assessments showed a statistically significant prolonged PIII and PV compared with controls, which improved in subsequent evaluations. Neuropsychological assessments across three years were normal, suggesting that altered auditory nerve central neurotransmission may be temporary and isolated, without affecting cognition.

Yilmaz et al.^25^ carried out BAEP testing in 22 neonates with hyperbilirubinemia up to age 12 months and found that two neonates had altered BAEP that suggested auditory neuropathy; this led to delays in language acquisition but no other neurological development alterations. On the other hand, two other subjects had neurological sequelae but normal BAEP results. It thus appears that neurological sequelae due to biliary pigment impregnation are selective.

Rhee et al.^10^ assessed auditory acuity following exchange transfusion in 11 neonates with severe hyperbilirubinemia, applying BAEP, TEOAE and immittance testing in their evaluation. These authors found normal immittance and TOAE recordings, but did not undertake a careful analysis of amplitudes at each frequency. In BAEP testing, electrophysiological thresholds were normal in seven neonates, high in one neonate and absent at 90 dBSPL in three neonates. Subsequent evaluations one year later revealed that electrophysiological thresholds improved considerably. BAEP alterations in these children with normal OAE test were interpreted as isolated retrocochlear damage with intact cochlear structures. One should be wary of such a conclusion, since detailed analyses of recorded emission amplitudes were not made in both groups.

Results of the present study underline the importance of OAE testing and auditory evoked potentials in the auditory evaluation of neonates, given that each of these tests assesses different sound stimulus transmission sites. Recordings should be analyzed quantitatively and in detail.

## CONCLUSION

Lower amplitudes in TOAE recordings and discretely prolonged PV and PI-V were found in the group of children with hyperbilirubinemia, suggesting that cochlear and retrocochlear auditory pathways may be affected. The importance of OAE and BAEP testing in these assessments and a careful quantitative and qualitative analysis of recordings is highlighted.
